# Land Development Rights, Spatial Injustice, and the Economic Development in Net-Incremental Reduction Regions of Construction Land: Evidence from Shanghai, China

**DOI:** 10.3390/ijerph20032560

**Published:** 2023-01-31

**Authors:** Jianglin Lu, Keqiang Wang, Hongmei Liu

**Affiliations:** 1 School of Public Economics and Administration, Shanghai University of Finance and Economics, Shanghai 200433, China; 2 Technology Innovation Center for Land Spatial Eco-Restoration in the Metropolitan Area, Ministry of Natural Resources, Shanghai 200003, China; 3 School of Finance and Business, Shanghai Normal University, Shanghai 200234, China

**Keywords:** construction land reduction, developing countries, economic development, land development rights, rural revitalization, spatial injustice

## Abstract

Rapid urbanization raises the issue of protecting development interests in net-incremental reduction regions of construction land (NRRCL). Spatial injustice (SI) is one of the key factors for the smooth implementation of construction land reduction (CLR) policies. This study theoretically analyzes the influence of SI on the economic development in CLR saving quota outflow regions, namely, NRRCL, and conducted empirical tests with the difference-in-differences model. The findings reveal that: (1) regional differences in CLR policy promote the transfer of land development rights from NRRCL to net-incremental increase regions of construction land (NIRCL) in economically developed regions, thus resulting in SI; (2) SI limits the economic development of NRRCL; (3) land-use planning negatively impacts economic development in planning reduced-type regions; (4) the off-site realization of spatial justice in the CLR process in suburbs has comparative advantages; (5) in the process of CLR, it is vital to promote the transfer of population from NRRCL to NIRCL to alleviate the negative impact of SI.

## 1. Introduction

The course of regional development in nations, such as China, that are considered “developing countries” is a major concern in economic research [1]. Several classic economic theories and models, such as Chenery’s models of urbanization and industrial growth [2,3], Harris and Todaro’s theory of economic development [4,5], Baumol’s theory of unbalanced growth [6,7,8], Lewis’s theory of binary economic structure [9], and Weber’s industrial location theory [10], have explored various aspects of economic growth and land development. They all basically follow incremental economic development theory—the economic development theory based on the expansion of construction land. However, rapid urbanization and industrialization lead to an increasing scarcity of available construction land, especially in developing countries. As “incremental planning”, characterized by construction land expansion, becomes unsustainable, the “stock” and “reduction” types of planning have attracted more attention from academic circles [11]. Moreover, as land resources have been overwhelmed by the extreme urban construction land expansion, people have become more aware of the consequences of urbanization and have become interested in sustainable urban development [12,13,14].

**Figure 1:**
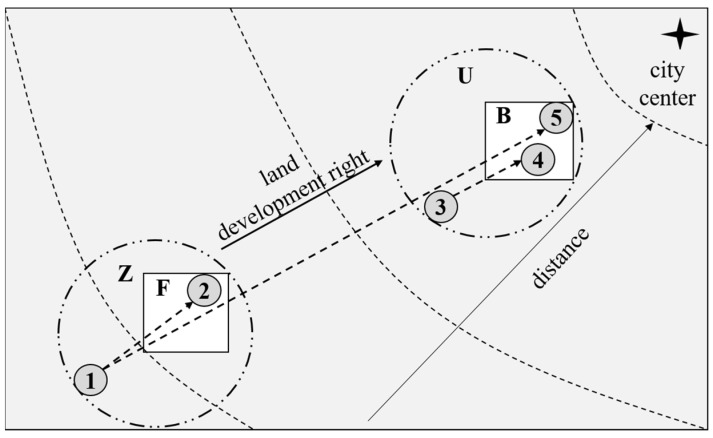
CLR and the transfer of land development rights.

Meanwhile, the suburbs of economically developed regions such as Shanghai and Beijing in China are—and will continue to be—in the rapid urbanization stage. It is necessary to understand how construction land quotas are determined. Construction land reduction (CLR) is a means of land consolidation [1]. It is helpful in solving the contradictions regarding supply and demand for construction land in economic and social development processes as a starting point to improve land use efficiency in economically developed regions [14]. Thus, it has been gradually promoted. CLR solves the contradiction between the supply and demand of construction land through the redistribution of land development rights. Land development rights are rights to develop land, namely, the right to use construction land, which is also an important facet of land property rights. The allocation of CLR is different from the traditional incremental land development rights. Traditional land development rights are formed by restricting land development and can call for converting agricultural land into construction land for development and utilization. Unlike the traditional and incremental land development rights, CLR produces a reduced type of land development right. That is, CLR first reclaims the inefficient construction land outside the centralized construction region, returning it to cultivated or ecological land, forming the saving quotas that can be equivalently transformed into the construction land quotas, then transfers the construction land quotas to the dominant regions (where the dominant regions can be within or cross the regions) to complete the transfer of land development rights (as shown in Figure 1). In Figure 1, from the city center to outward, there are two regions of U and Z. Among them, region U is adjacent to the urban center, and there is a centralized construction region B within region U, while region Z is far away from the urban center, and there is a centralized construction region F in region Z. CLR will reclaim the inefficient construction land (such as Plot 1 in region Z and Plot 3 in region U) outside the centralized construction regions into cultivated land or ecological land, and then allocate the saving quotas to the efficient construction regions (such as Plot 2 in the centralized construction region F, or Plot 4 in the centralized construction region B), which represents the transfer of land development rights within region U and region Z. Also, it is possible that after the reduction of Plot 1, the saving quotas will be used for Plot 5, which is the cross-regional transfer of land development rights.

While CLR addresses the tight constraints of the construction land quota in China’s urban development process, it also limits the development of net reduction areas of CLR [1], leading to a lack of spatial justice (SJ) [15]. SJ and the development of net-incremental reduction regions of construction land (NRRCL) under the control of the total amount and intensity of construction land requires increased attention. Soja links justice to other broad concepts referring to the qualities of a just society [16]. Since Rawl’s concept of distributional justice was introduced [17], the distribution of resources has been applied to rural SJ [18]. SJ is the internal basis for the legitimacy and rationality of urban development in China and is one of the main goals of urban planning [19]. From the perspective of CLR, the reduction mainly occurs in regions with poor location conditions and low construction land output efficiency [20]; these regions are considered NRRCL. The cultivated land occupation–compensation balance quotas and space development balance quotas generated by CLR (referred to as the saving quotas) are mainly used in net-incremental increase regions of construction land (NIRCL) with high output efficiency and good location conditions. This cross-regional transfer of land development rights induced by CLR results in spatial injustice (SI), restrains the realization of those rights for NRRCL and reduces their existing land development rights. Furthermore, the SI issue of NRRCL is increasingly common and affects the implementation of CLR policy.

SI restrains the play of the advantage of backwardness of NRRCL, thus affecting its development. The control of the total amount and intensity of construction land restricts the expansion of urban and non-agricultural industries. Therefore, according to Lewis’ theory of binary economic structure [9], the development of NRRCL cannot be realized, and, in turn, the transformation of a binary economic structure to a single economic structure becomes limited. As a result, if people in NRRCL do not support CLR policies, the economic development of NRRCL cannot be well realized [1]. Eventually, the whole region would fail to develop. Thus, it enters a “static” state of economic development such that the potential growth is 0. Hence, it is necessary to identify the impact mechanisms of SI and clarify the effects on the economic development of NRRCL during the CLR process.

The problem of slow development in NRRCL is one of the constraints that limit the high-quality development of CLR regions. It is of great practical significance to correct SI in CLR and to promote the realization of land development rights and backwardness advantages of NRRCL. What are the theoretical mechanisms by which SI influences the economic development of NRRCL? Do the available data support this theoretical mechanism? How can the negative impacts of SI on the economic development of NRRCL be corrected? We have yet to find answers to these questions in the literature. Since there are few cities that have implemented CLR on a region-wide scale, and the relevant data are difficult to obtain, there are few quantitative studies on CLR in the existing literature, especially for the development of NRRCL from the perspective of SI caused by the transfer of land development rights. Therefore, this study analyzes the impact of SI on the economic development of NRRCL. We first analyze the mechanism of SI affecting the economic development of NRRCL and propose five research hypotheses. Then, based on the data from W District, Shanghai, China, we empirically test the mechanism by which SI affects the economic development of NRRCL through the difference-in-differences (DID) model. Finally, we propose policy implications for solving the CLR issues related to SI so the economic development of NRRCL can be realized.

The study is structured into six sections. Following the introduction in Section 1, the literature will be reviewed and presented in Section 2. Section 3 describes the materials and methods, followed by the results in Section 4. Section 5 presents the discussion. Finally, we present policy implications and major conclusions in this study.

## 2. Literature Review

Agricultural land fragmentation and land consolidation are widespread in countries all over the world and achieving better development through land consolidation has attracted attention. Fragmentation of agricultural land reduces the efficiency of all aspects of agriculture, such as increased production cost and reduced yield, revenue, profitability, and efficiency [21]. Industrial land and rural residential land have the highest degree of fragmentation, and the shortage of construction land requires gradually reducing the fragmentation of construction land [22]. The economic and social declines in rural regions have intensified in Eastern and Western European countries over the past two decades [23]. Land consolidation has become a tool for improving the efficiency of land cultivation and supporting rural development [24,25,26], improving land productivity, and even improving the total factor productivity through technological progress [27]. Consolidation solves the problem of land fragmentation through the planning of adjustments to land ownership [26] to form a larger scale of land ownership and a more reasonable layout of land ownership patterns [23,28]. In Turkey, land consolidation is used to improve rural development [29]. In Western Europe, land consolidation is part of a broader rural development plan, including improving agricultural production, employment, and taxation, while protecting natural resources and the environment [23,25,29]. In China, land consolidation is also used as a means of improving agricultural production conditions, improving economic efficiency and achieving sustainable development [27,30,31].

Urbanization is a dynamic and multi-dimensional development process [32], often accompanied by the expansion of construction land. With unprecedented urbanization, the shortage of construction land has become the main factor restricting further economic and social development. CLR is a tool of land consolidation [1]. Through CLR, we can realize the optimization of the utilization structure of construction land to meet the demand of economic and social development for construction land and solve the contradiction of insufficient construction land in the process of development without increasing the total amount of construction land [20,33]. Existing studies generally perceive the approach of CLR as an important means of controlling the expansion of construction land and achieving sustainable development [14,20,34]. Several studies have focused on CLR’s effects and the benefits of different actors, such as industrial land reduction policy’s impacts on town- and village-level interests [14] and the full life-cycle management mechanisms and policies regarding industrial land [35]. There are also studies that have examined residents’ selection behavior of compensation schemes for CLR [1].

Relevant studies on SI and SJ are mainly qualitative studies. “Justice” may be interpreted by fairness and equality [36]. Fairness is an abstract social and political concept [37], and both fairness and justice include addressing social, environmental, and economic justice and equity in all developed or developing countries [38]. Justice is also a primary social value [39]. More specifically, the concept of fairness is the fundamental social aspect of sustainable development [40], including recognition, redistribution, and fair participation [38]. The logical thread of capital criticism runs through all the works of Harvey, such as Social Justice and the City [41], The Limits to Capital [42], The Urbanization of Capital [43], etc. Spatial fairness means that the allocation between services and each resident’s needs, preferences, and service standards is average [44]. Justice is one of the main goals of planning ideas, which is also widely accepted by scholars of all periods [19]. The existence and implementation of land-use planning, urban planning, and territorial space planning cause SI, such as in issues in the planning of urban public space [45,46].

In summary, established studies have mainly studied the economic and social impacts of CLR, and less research has been conducted on the regional development imbalance caused by CLR. Moreover, the existing studies on SI or SJ are mainly some qualitative studies. In the process of CLR, we cannot yet find the answer from the existing literature about how the SI of CLR affects the economic development of NRRCL. Shanghai was the first province in China to implement CLR policies on a region-wide scale [14], and it has a relatively well-developed CLR policy process [1]. Therefore, this study takes W district, Shanghai, China as an example, to study the impact of SI on the economic development of NRRCL. The possible innovations of this study are: (1) it constructs a theoretical model to study the theoretical mechanism of SI; (2) it explains the influencing mechanism of SI on NRRCL economic development from the perspective of land development rights and advantages of backwardness; (3) five research hypotheses were tested empirically.

## 3. Materials and Methods

### 3.1. Theoretical Analysis and Research Hypothesis

Based on a simple theoretical model, the development dilemma of NRRCL under the control of the total amount and intensity of construction land was analyzed. Five research hypotheses are proposed for SI affecting the economic development of NRRCL based on the cross-regional transfer of land development rights, backwardness advantages, land use planning, and population transfer.

#### 3.1.1. Basic Theoretical Model

First, we analyze the production department.

Assume that there are several representative manufacturers and workers in region U and region Z who can flow freely between cities [47]. Land, similar to labor and capital, is also a basic factor of production [48], and representative manufacturers employ workers, as well as lease capital and land for production in competitive factor markets. Besides labor, capital, and land, manufacturing is also affected by agglomeration levels and government preference [49,50,51]. Under the planned land-use control, the allocation of construction land is in the form of “reduction to increase” [1]. Supposing that the quotas of increased construction land in region U and region Z are equal to the reduced construction land area.

Drawing on existing research [47,52], this study introduced construction land and material capital elements to characterize the above research assumptions with a simplified production equation: in region *i* (*i* = U,Z), there are *m_i_* representative manufacturers, the labor, capital and construction land input of each manufacture is respectively *l_i_*_,ave_, *k_i_*_,ave_ and *s_i_*_,ave_, the public expenditures of local governments is *g_i_*. Assuming the production function of the representative manufacturer is the Cobb–Douglas production function, then the yield *y_i_* of each manufacturer in region *i* is as below:

*y_i_* = *A_i_m_i_^εi^*(*l_i_*_,ave_*)^αi^*(*k_i_*_,ave_)*^βi^*(*s_i_*_,ave_)*^ηi^g_i_^φi^* (1)

In Equation (1), *A_i_* refers to the level of production technology in region *i*, and *ε_i_* refers to the effect of manufacture agglomeration in region *i*. The positive externalities generated by the agglomeration of production factors are the main reasons for the stronger urban production capacity and faster economic growth [47]. Agglomeration can produce a scale effect and greater labor market demand [53,54,55]. Therefore, we set *ε_i_* > 0. *α_i_*, *β_i_*, *η_i_*, and *φ_i_* refer to the output elasticity of the labor force, the output elasticity of the capital, the output elasticity of the construction land, and the production externalities of the government expenditures in region *i*, respectively (0 < *α_i_*,*β_i_*,*η_i_*,*φ_i_* < 1). As for each region, the analysis idea is the same, so we ellipsis the subscript *i*.

The total yield of manufactures in region *i* is:

*Y = ∑y* = *Am*^1+^*^ε^*^−^*^α^*^−^*^β^*^−^*^η^l*^α^*k^β^s^η^g^φ^* (2)

In Equation (2), *l* = *ml_i_*_,ave_ refers to the total labor input in region *i*, *k* = *mk_i_*_,ave_ refers to the total capital input of manufacturers in region *i*, and *s* = *ms_i_*_,ave_ refers to the total construction land input of manufactures in region *i*.

In reality, manufacturers are rational manufacturers, and follow the market principles. According to Weber’s theory of industrial location, the minimum cost is the basic factor in business location [10]. Cost here includes transportation cost and land rent. That is, manufacturers conduct location layout analyses according to the transportation cost and land rent [56]. Assuming the rental rate of construction land for manufactures is *R_m_*, and the commuting cost for manufactures is *C_m_*, then, manufacturers organize production according to profit maximization, and the problem is to maximize profit (*Pro*) at a given wage rate *w*, capital interest rate *r*, construction land rent rate *R_m_* and commuting cost *C_m_*:

*Max Pro* = *Am^ε^*^−*α*−*β*−*η*^*l*^α^*k^β^s^η^g^φ^* − *wl_i_*_,ave_ − (*r* + *δ*)*k_i_*_,ave_ − *R_m_s_i_*_,ave_ − *C_m_* (3)

In Equation (3), *δ* refers to the capital depreciation rate. In equilibrium, the wage rate and the construction land rent rate represent the marginal return rate of the elements, and the marginal output of material capital is the sum of the capital interest rate and the depreciation rate. The marginal product of labor is:

*w* = *Aαm*^1+*ε*−*α*−*β*−*η*^*l^α^*^−1^*k^β^s^η^g^φ^* (4)

The marginal product of capital is:

*r* + *δ* = *Aβm*^1+*ε*−*α*−*β*−*η*^*l^α^k^β^*^−1^*s^η^g^φ^* (5)

The construction land rental rate is equal to the marginal output of construction land:

*R_m_* = *Aηm*^1+*ε*−*α*−*β*−*η*^*l^α^k^β^s^η^*^−1^*g^φ^* (6)

*Pro* is the rest of the manufacturing output minus the wage cost, capital cost, construction land rent, and commuting cost. Then:

*Pro* = (1 – *α* – *β* − *η*)*Am^ε^*^−*α*−*β*−*η*^*l^α^k^β^g^φ^s^η^* − *C_m_* (7)

Second, we analyzed the urban structure.

Further, we consider the impact of the transportation and land rent time value. During urban operation, local governments prefer to obtain fiscal revenue from land rent. According to the standard assumption [52,57,58], there is only one city center; the city extends from the center to the periphery, showing a standard circular urban structure, and the city center is a centralized construction region, where each worker and manufacturer occupies one unit of land area. It is assumed that the land is homogeneous (land homogeneity is relative to the carrying function of land: the natural factors of land are the same, the conditions of construction land are the same), manufacturers and workers are rational, and they choose the best location according to the transportation cost and land rent with perfect mobility [56]. There are *m* manufacturers occupying the centralized construction regions with convenient conditions, with *l* workers living outside the centralized construction regions (due to the higher requirements for transportation and information exchange, the closer to the urban region, the higher the quality of the population, the greater the technical advantages and production efficiency, the lower the transportation cost, and the higher the cost of living. Hence, the manufacturers are located in the center of the circle, while the workers are located in the outer ring [47]). Setting the radius of the centralized construction region evenly distributed by *m* manufacturers as *D*_0_, and the radius of the whole city as *D*_1_, we have:

*D*_0_ *=* ∫[0,*D*_0_]2*πudu = m* (8)

*D*_1_*= ∫*[0,*D*_1_]2*πvdv = m + l* (9)

Then, we have *D*_0_ = *π*^−1/2^*m*^1/2^, *D*_1_ = *π*^−1/2^(*m* + *l*)^1/2^.

Each manufacturer can choose the location freely in the centralized construction region, and each worker can choose the living place outside the centralized construction region. However, no matter how the manufacturers and workers “choose the location”, in equilibrium, the “commuting cost + land rent” of each manufacturer should be always equal, and the “commuting cost + land rent” of each worker should also be always equal [52]. Since manufacturing is located in the centralized construction region and the residents live in the periphery of the centralized construction region, the commuting cost per unit distance of manufactures is set as *ψ_m_* and the commuting cost per unit distance of workers is set as *ψ_l_*, then *ψ_m_* < *ψ_l_*. The commuting cost for manufactures located at the very edge of the centralized construction region is *ψ_m_D*_0_. If we standardize the rent of construction land here to 0 [57], then in equilibrium, the total expenditures of the other representative manufacturers are all *ψ_m_D*_0_. The equilibrium characteristic of the construction land market lies in the rent gradient. It extends from the center of the centralized construction region to the edge of the centralized construction region with a distance of *D*_0_ away from the center of the centralized construction region. Then, the rent of a representative manufacturer with the distance to the center of the centralized construction region of *x* is *R_x_* = *ψ_m_*(*D*_0_ − *x*); then, the total land rent for all manufactures *TR_m_* is:

*TR_m_* = ∫[0,*D*_0_]2*πxR_x_dx* = (*π*^−1/2^*ψ_m_m*^3/2^)/3 (10)

The total land rent for all manufacturers *TE_m_* is *mψ_m_D*_0_, that is *π*^−1/2^*ψ_m_m*^3/2^. Then, the total commuting cost for all manufacturers *TC_m_* is:

*TC_m_* = *TE_m_* − *TR_m_* = *π*^−1/2^*ψ_m_m*^3/2^ − *TR_m_* = (2*π*^−1/2^*ψ_m_m*^3/2^)/3 (11)

Similar to the analysis of manufacturers’ commuting costs and land rent, the total commuting cost for all workers living from *D*_0_ to *D*_1_ is *TC_l_*:

*TC_l_* = ∫[*D*_0_,*D*_1_]2*πψ_l_t*^2^*dt* = 2*ψ_l_π*^−1/2^[(*m* + *l*)^3/2^ − *m*^3/2^]/3 (12)

Similar to the calculation method of the total expenditure of manufacturers, the total expenditure of workers *TE_l_* is *lψ_l_D*_1_, that is, *lψ_l_*π^−1/2^(*m* + *l*)^1/2^. Then, the total land rent cost of workers *TR_l_* is:

*TR_l_* = *TE_l_* − *TC_l_* = *lψ_l_π*^−1/2^(*m* + *l*)^1/2^ − *TC_l_* = *lψ_l_*π^−1/2^(*m* + *l*)^1/2^ − 2*ψ_l_*π^−1/2^[(*m* + *l*)^3/2^ − *m*^3/2^]/3 (13)

Third, we analyzed the government preference for construction land without constrained construction land planning quotas.

The government should provide public services and develop the local economy, which depends on tax revenues. The city government collects land rent as fiscal revenue for public sector expenditure such as for education, health care and transportation. It invests in infrastructure construction to improve the local production environment, attract manufacturers to increase capital inflow [47], and promote the local economic development.

For simplification, assume that in addition to collecting the construction land rent from manufactures and workers as a source of income, the government receives *θ* times the income of the land rent generated by the other ways. The government maximizes the income by selecting the amount of manufactures *m*, workers *l*, public expenditure *g*, and subsidizes the reduced manufactures and workers [57]. The subsidy levels are *Sub_m_* and *Sub_l_* (the subsidy level here includes the government subsidies to manufacturers’ and workers’ losses due to CLR), respectively, which enables the manufacture profit *Pro* and worker income *Inc* to be not lower than the average level of *Pro*_ave_ and *Inc*_ave_ of the whole society, respectively. The optimization problem is as below:

*Max* (1 + *θ*)(*TR_m_* + *TR_l_*) − *mSub_m_* − *lSub_l_* − *g* *s.t. Sub_l_* + *w* − *ψ_l_π*^−1/2^(*m* + *l*)^1/2^ = *Inc*_ave_; *Sub_m_* + *Pro* − *ψ_m_π*^−1/2^*m*^1/2^ = *Pro*_ave_ (14)

The solution is:

*Sub_m_* = [6*Ω* + *Ψ* + 3*θ*(2*Ω* + *Ψ*)]*m*^1/2^/4 + 3*Ω*[*θl*(*m* + *l*)^−1/2^ − 2(1 + *θ*)(*m* + *l*)^1/2^]/4 + *A*[*α* + (1 – *β* − *η*)(*ε* – *α* – *β* − *η*)]*m^ε^*^−*α*−*β*−*η*^l^α^k*^β^s*^η^g*^φ^; *Sub_l_* = 3*θΩl*(*m* + *l*)^−1/2^/4 + [2(*α* − 1) + *β* + *η*]*αAm*^1+*ε*−*α*−*β*−*η*^*l^α^*^−1^k*^β^s*^η^g*^φ^; g* = [*φ*(1 – *β* − *η*)*Am*^1+*ε*−*α*−*β*−3*η*/2^*l^α^*^+*β*+*η*^*β^β^ω^β^*^+*η*^*η^η^*/(*r* + *δ*)*^β^α^β^Ψ^η^α^η^*]^1/(1−*φ*)^; k* = *lβω*/[*α*(1 + *ε* – *α* – *β* – *η* + *δ*)]; s* = 3*ηlωπ*^1/2^/(*ψ_m_m*^1/2^*α*) (15)

In Equation (15), *Ψ* = 2*π*^−1/2^*ψ_m_*/3, *Ω* = 2*π*^−1/2^*ψ_l_*/3. According to Equation (15), the optimal configuration area of the construction land in region *i* is:

S_i_* = *m_i_*⋅(3*η_i_l_i_ω_i_π*^1/2^)/(*ψ_i_*_,*m*_*m_i_*^1/2^*α*_i_) = *m_i_*⋅3*η_i_*⋅(1/*α_i_*)⋅(*l_i_w_i_m_i_*)⋅[1/(*π*^−1/2^*ψ_i_*_,*m*_*m_i_*^3/2^)] (16)

In Equation (16), *ψ_i_*_,*m*_ is the commuting cost per unit distance of manufacturers in region *i*. Under the condition wherein construction land quotas are not a constraint, the optimal configuration area of construction land in region *i* is positively proportional to the number of manufacturers *m_i_* in region *i*, the productivity level of construction land *η_i_* and the total wage of labor *l_i_w_i_m_i_* (the total wage of labor represents the total wage or output scale of the labor force in the region, and also reflects the value created by the employment of manufacturers in the region). It is inversely proportional to the output level of labor *α_i_* (The higher the output level of workers in the region, the less the number of workers is needed) and the total expenditure level of manufacturers *π*^−1/2^*ψ_i_*_,*m*_*m_i_*^3/2^.

Fourth, we analyzed the marginal space reduction tendencies for construction land and the marginal space use tendency of land development rights under the control of construction land planning quotas.

The unbalanced development strategy under the control of the total amount and intensity of construction land requires the construction land quotas to gather in advantageous regions [59]. Due to the control, the total area *S_0_* of new construction land in regions U and Z is extremely limited; that is. *S_U_* + *S_Z_* ≤ *S_0_*. According to the theoretical derivation of Equations (16) and (2), if assuming that except for the productivity level *β_U_* > *β_Z_* of the construction land, regions U and Z have exactly the same conditions, that is, other factors such as labor and capital are sufficient and not different, then we have S_U_* > S_Z_*, and Y_U_* > Y_Z_* (“*” represents that the final quotas are achieved). Therefore, expanding the construction land in region U is an inevitable choice to achieve the development goals of the whole region.

The central government requires the implementation of the control of the total amount and intensity of construction land, and the construction land quotas are often coordinated in a larger scope, which is essentially unbalanced development [59]. The main subject of CLR is the township, while the saving quotas are used by the district level, or even by the municipal level. The larger the spatial radius of the construction land quotas allocation, the greater the potential for efficiency improvement. The construction land quotas in Figure 1 are transferred from Plot 1 to Plot 5. Limited construction land-use quotas are allocated to regions with higher efficiencies and better locations. In the process of saving quotas’ allocations, NRRCL can allocate less construction land. Therefore, during the process of CLR, the marginal space reduction tendency (the government’s tendency of reduction location, reflecting whether the government prefers to conduct CLR in low-efficiency regions or to conduct CLR in high-efficiency regions) of inefficient construction land owned the government is in the low-efficiency regions, while the marginal space use tendency (the government’s allocation tendency for saving quotas formed after CLR, reflecting whether the government is more inclined to allocate the saving quotas obtained by CLR in low-efficiency regions or in high-efficiency regions) of land development rights is in high-efficiency regions. Studies have found that the tax amount per unit area in the development zone is three times or even five times that outside the development zone [20]. The development zone belongs to the centralized construction region, so the centralized construction region belongs to the high-efficiency region and is the growth point of the construction land. Regions outside the centralized construction region belong to the low-efficiency region, which is the focus of CLR.

For region Z, during the CLR process, the inefficiency of Plot 1 outside the centralized construction region F is reduced and reclaimed for cultivated land or ecological land. Part of the saving quotas is used for Plot 2 in the centralized construction region F; the other part is used for Plot 5 in the centralized construction region B of region U. Overall, for region Z, the saving quotas are greater than the construction land use quotas, thus becoming NRRCL. For region U, during the CLR process, the inefficient Plot 3 outside the centralized construction region B is reduced and reclaimed into cultivated land or ecological land, and the saving quotas are all used for Plot 4 in the centralized construction region B. Overall, for region U, the saving quotas are less than the construction land use quotas, thus becoming NIRCL. This explains the cross-regional transfer of land development rights; see Figure 1. Under the public ownership condition, the initial distribution of land development rights is equal across all the people in each region. For example, people in region U have land development rights in region U, and people in region Z have land development right in region Z. Therefore, they benefit from the realization of land development rights, which they are striving to obtain. When the total increment of construction land is controlled, the net increase in opportunity of construction land is obtained through competition. When the total increment of construction land is zero, each tries to achieve the least reduction and the greatest increases as possible through competition. The CLR process is the process of regions U and Z striving for less reduction and more increment. People in each region are working for their own development interests. In the process of CLR, when the reduced area of inefficient construction land in region Z is greater than the newly added area of the construction land, its residents will claim for paid transfer. According to Coase Theorem, the clearer the property rights, the more reasonable the transfer, and the easier the redistribution of land development rights becomes [60,61]. Unreasonable or even free transfer of land development rights will inevitably affect the enthusiasm of CLR and the transfer of land development rights in region Z.

W.A. Lewis, the winner of the 1979 Nobel Prize in Economics, studied economic development under an unlimited supply of labor. Moving surplus labor from the agricultural sector to productive posts in the modern urban sector contributes to economic development [9]. Lewis’s theory of binary economic structure is based on the premise of an unlimited supply of construction land, and the labor force in the agricultural sector is transferred to the non-agricultural sector due to the wage difference. However, due to the total amount of construction land control, the construction land cannot be supplied indefinitely. CLR finds a new way through the transfer of construction land quotas, that is, the transfer of rights for development under the tight constraints of construction land quotas. CLR also involves the reconfiguration of the saving quotas. Due to the fixation of land location, CLR is, essentially, the reconfiguration of land development rights under the planning constraints. The saving quotas obtained through CLR in NRRCL are allocated to NIRCL.

In 1947, the concept of “land development rights” was first proposed in Britain’s Urban and Rural Planning Law. Later, it was adopted by the United States, Germany, France, Japan, and other developed market economy countries. The United States Development Rights Transfer Program is a multi-objective policy tool for planners to achieve a broad range of planning goals [62,63]. Ronald H. Coase (the 1991 Nobel Prize winner in Economics) believed that property rights are essential for economic growth [60,61]. The Coase Theorem emphasizes the importance of defining property rights, and property rights’ institutional arrangements with relatively low transaction costs should be sought to improve the overall benefits. Because the marginal space use tendency of land development rights is used for the peri-urban regions, land development rights in the remote suburbs are difficult to realize. Such an unbalanced growth strategy often leads to an uneven allocation of construction land [20]. Development rights for cultivated lands reclaimed after CLR are also difficult to realize, and land development rights are used for NIRCL across regions. Under the premise of the control of the total amount and intensity of construction land, the use of planning space needs to be moved through CLR. Similar to land ticket trading in Chongqing, China, CLR can bring new arable land to farmers and provide incremental construction land quotas for industrial and urban scale development. This spatial transfer of land development rights under CLR causes SI and frequent conflicts of interest [11] that restrict the economic and social development of NRRCL [20]. Compared with NIRCL, NRRCL has reduced development rights, and its development potential is limited [1,20]. Such transfers of land development rights may also limit the advantage of backwardness for NRRCL. The cross-regional transfer of land development rights during the CLR process limits the realization of land development rights in the NRRCL. It promotes the economic and social development of NIRCL but restricts the economic and social development of NRRCL. Thus, Hypothesis 1 is proposed:

**Hypothesis 1 (H1).**  *The cross-regional transfer of land development rights caused by CLR produces SI, which may lead to the decreased enthusiasm of NRRCL for CLR*.

#### 3.1.2. SI, Backwardness Advantages, and the Economic Development of NRRCL 

Equal access to basic public facilities is an important manifestation of the SJ [64]. A certain population requires certain numbers and types of services [65,66,67], which requires certain land bearing [68]. Although the balanced allocation of the saving quotas leads to low economic efficiency, cross-regional reallocation of construction land use rights is not conducive to total GDP growth in underdeveloped regions. SI results in an insufficient allocation of construction land quotas; allocation is not increased, and can even be decreased in the NRRCL, which is unfavorable to its industrial development.

In 1962, Alexander Gerschenkron proposed a theory of the advantage of backwardness. In subsequent papers, he pointed out that relatively backward industrialized countries (such as Germany) had many significantly different industrialization processes and characteristics from those of advanced countries (such as the United Kingdom) [69]. However, although late-comers tend to grow faster than developed countries, they tend to face an economic slowdown when they approach the forefront of technology [70]. Moreover, the further a country is from the global frontier of science and technology, the more it can gain from this advantage of backwardness [71].

The development history of the world economy is the development history of backward countries catching up with advanced countries. Countries with relatively backward development can find creative alternatives to the preconditions of industrialization and achieve the same or similar industrialization effect. Countries with relatively backward development also have access to advanced countries’ funds, technology, and equipment and learn from their advanced management experience to achieve economic catch-up; thus, they can enter a higher stage of industrialization in a relatively shorter time. The advantage also applies to manufacturers [72].

SI limits the realization of land development rights and constrains NRRCL’s advantage of backwardness, which is detrimental to NRRCL’s economic development. Land-use conflicts occur because the restriction of land spatial allocation for the advantage of backwardness is ignored [73].

Based on the above analysis, Hypothesis 2 is put forward:

**Hypothesis 2 (H2).**  *SI restricts the play of the advantage of backwardness mechanism and thus limits the economic development level of NRRCL*.

Different from the property rights relationship under Western private ownership, China implements public ownership of land rights. Western cities such as New York and London rely on property right relationships, which, for landowners, include the right to build, buy, mortgage, and lease [74]. However, under the premise of public land ownership in China, urban development space, that is, construction land allocation to cities and non-agricultural industries, should strictly follow spatial planning, use control, and planning management. The control of the total amount and intensity of construction land strengthens the implementation of construction land planning. Since 2014, Shanghai, China, has worked out the “country unit village planning” at the township level and the “detailed control planning” process. In the process of making these plans, an important task is to divide villages into three categories, including protecting villages, retaining villages, and withdrawing villages, and plan layouts for the industrial land according to the decomposed construction land quotas from top to bottom to specify the location and area of the reduction regions. The policy was revised several times from then on, and the name of the plan has changed, but in 2017, it was connected to the Master Plan and General Land Use Plan of W District, Shanghai (2017–2035). With the formation of the planning scheme, the increased and decreased attributes of the corresponding location, the possibility of industrial expansion, and the possibility of village retention all become clear. The planning played a guiding role in economic development, land use structure allocation, employment, investment, and so on. According to the Master Plan and General Land Use Plan of W District, Shanghai (2017–2035), W District is divided into three categories, namely, a planning increment-type area, planning balance-type area, and planning reduction-type area. Compared with planning increment-type regions, land-use planning has a negative impact on the economic development of the other two types of areas. Accordingly, Hypothesis 3 is proposed.

**Hypothesis 3 (H3).**  *Land-use planning may negatively impact economic development in the regions under plans for balanced or decreased development*.

Under the premise of the control of the total amount and intensity of construction land, it is very difficult to fully exercise land development rights locally. During the saving quotas transfer, the total factor productivity of NIRCL is improved through structure optimization, which contribute to protecting the development rights and interests of NRRCL. Specifically, the implementation of SJ of NRRCL has two paths: in situ realization and off-site realization. The former means that both land development rights and saving quotas formed through CLR are used for the economic development in NRRCL. Additionally, NRRCL can get assistance from NIRCL in the concentration of the residence and employment (on the one hand, the transfer of an NRRCL population to NIRCL improves the welfare of the transferred population, and thus benefits the transferred population; on the other hand, the total population of NRRCL decreases, which helps to improve the welfare level of the remaining population) between their populations or economically equivalent compensation. Due to NIRCL’s comparative advantages over NRRCL in technology, capital, location, and social security, in situ realization of NRRCL’s land development rights is inferior to off-site realization. Therefore, Hypothesis 4 is proposed.

**Hypothesis 4 (H4).**  *Compared with the in situ realization of SJ in CLR, the off-site realization of SJ in the CLR process in suburbs has some advantages*.

#### 3.1.3. The Impact of NRRCL’s Population Transfer on Economic Development

Assume the natural population growth rate of a region is zero, and all the other mechanical population growth rates except CLR are zero (according to the 2021 Statistical Yearbook of W District and the 2016 Statistical Yearbook of W District, the registered population of W District was 525,204, of which the birth population was 2238 and the death number was 4286. The natural growth rate of the registered population was about negative 0.0039. The immigration and emigration numbers were 1914 and 191, respectively. The mechanical growth rate of the population is about 3.3‰. In 2016, the registered population was 520,404, and the average annual growth rate from 2016 to 2020 was about 2.30‰). Assume that the GDP in a region is produced entirely by non-agricultural industries (according to the 2021 Statistical Yearbook of W District, the total agricultural output value in 2020 is only 2.2358 billion CNY, and the regional GDP is 107.63 billion CNY. It can be seen that the total agricultural output value accounts for only 2.08% of the regional GDP). The *GDP_t_* of year *t* is mainly from construction land. Therefore, CLR will affect GDP.

The relationship between the *GDP_t_* and the administrative division area (*S_AD_*_,*t*_), GDP per unit of administrative division area (*Land_AD_GDP_t_*), is as Equation (17):

*GDP_t_* = *S_AD_*_,*t*_⋅*Land_AD_GDP_t_* (17)

According to Equation (17), we have:

*Land_AD_GDP_t_* = *GDP_t_*/*S_AD_*_,*t*_ (18)

Since *S_AD_*_,*t*_ remains the same, *Land_AD_GDP* and GDP show changes in the same direction. If the population transferred along with CLR, assuming the area of the construction land that year was *S_CL_*_,*t*_, the total population is *Peop_t_*, and supposing that the productivity of the construction land is unchanged, the CLR saving quota transfer amount for NRRCL is *Z_trans_*_,*t*_, and the amount of population transfer to the NIRCL is *Peop_trans_*_,*t*_, the relationship between the *GDP_t_* and *Peop_t_*, *S_CL_*_,*t*_, *S_AD_*_,*t*_, GDP per capita (*PerGDP_t_*), GDP per unit of construction land area (*Land_CL_GDP_t_*) and *Land_AD_GDP_t_* is as Equations (19) and (20):

*GDP_t_* = *Peop_t_*⋅*PerGDP_t_* (19)

*GDP_t_* = *S_CL_*_,*t*_*⋅Land_CL_GDP_t_* (20)

*S_CL_*_,*t*_ and *Peop_t_* of year *t* in NRRCL are respectively shown in Equations (21) and (22):

*S_CL_*_,*t*_ = *S_CL_*_,0_ − *∑Z_trans_*_,*i*_ = *S_CL_*_,0_ − *t*⋅*Z_trans_*_,ave_ (21)

*Peop_t_* = *Peop*_0_ − *∑Peop_trans_*_,*i*_ = *Peop*_0_ − *t*⋅*Peop_trans_*_,ave_ (22)

In Equations (21) and (22), *i* ∈ 1, 2, …, *t*. *S_CL_*_,0_, *Peop*_0_ respectively represent the construction land amount and population at the early stage of NRRCL. *Z_trans_*_,ave_, *Peop_trans_*_,ave_ respectively represent the average construction land transfer and population transfer of NRRCL. By Equations (17), (19) and (22), we have:

*PerGDP_t_* = S*_AD_*_,*t*_⋅*Land_AD_GDP_t_*/*Peop_t_* = 1/(1 − *t*⋅*Peop_trans_*_,ave_/*Peop*_0_)⋅*Land_AD_GDP_t_*⋅*S_AD_*_,*t*_/*Peop*_0_ (23)

Since *S_AD_*_,*t*_/*Peop*_0_ is constant, according to Equation (23), the population reduction in NRRCL contributes to the increase in *PerGDP_t_*.

Since the GDP of a region is produced entirely by non-agricultural industries, then the output of construction land is the output of the administrative region,

*Land_AD_GDP_t_* ∝ *Land_CL_GDP_t_*, and we further gain Equation (24):

*PerGDP_t_* = *S_AD_*_,*t*_⋅*Land_AD_GDP_t_*/*Peop_t_* ∝ *S_CL_*_,*t*_⋅*Land_CL_GDP_t_*/*Peop_t_* =(S*_CL_*_,0_ − *t*⋅*Z_trans_*_,ave_)⋅*Land_CL_GDP_t_*/(*Peop*_0_ − *t*⋅*Peop_trans_*_,ave_) =[(1 − *t*⋅*Z_trans_*_,ave_/*S_CL_*_,0_)/(1 − *t*⋅*Peop_trans_*_,ave_/*Peop*_0_)]⋅*Land_CL_GDP_t_*⋅*S_CL_*_,0_/*Peop*_0_ (24)

Along with the CLR process, the inefficient construction land is reduced, leaving the overall efficiency of the construction land on the rise; this is *Land_CL_GDP_t_*, which presents a rising tendency. If *S_CL,0_*/*Peop_0_* is constant, then according to Equation (24), the major factor influencing the decrease and increase of *PerGDP_t_* is (1 − *t*⋅*Z_trans_*_,ave_/*S_CL_*_,0_)/(1 − *t*⋅*Peop_trans_*_,ave_/*Peop*_0_). When the elasticity of population transfer is less than that of construction land quotas, *PerGDP_t_* will decrease. Therefore, when the productivity of labor and land does not increase, maintaining the per capita GDP of the retained population of NRRCL requires the population of NRRCL to reduce along with the decrease of construction land quotas. If the rate of population transfer is lower than the transfer rate of construction land quotas, the per capita GDP decreases. Therefore, Hypothesis 5 was put forward.

**Hypothesis 5 (H5).**  *Land_AD_GDP and GDP change in the same direction during CLR. The negative impact of SI on GDP per capita is greater than it is on total GDP or GDP per unit of administrative division area*.

### 3.2. Research Region Selection

In this study, W district, Shanghai was selected as the sample for analysis. The reasons for this are as follows. First, in China, Shanghai was the first provincial city to implement CLR policies on a region-wide scale [14]. Shanghai has a relatively well-developed CLR policy process [1]. Second, in 2014, W District, Shanghai took the lead in implementing the policy of CLR to pursue the high-quality use of land. Third, CLR mainly occurs in the suburbs of the city, and W District of Shanghai is one of the suburbs. Therefore, it can meet the requirements of our analysis of CLR policy. Fourth, the land-use data of administrative villages or residents’ committees were extracted from the land-use status of each year with ArcGIS. Such vector data at the administrative villages or residents’ committees level are extremely difficult to obtain. The author could only obtain the land-use status map of W District. Besides, at the village level, some villages belong to the NRRCL, while some villages belong to the NIRCL. W District can reflect the actual situation of CLR in Shanghai, China. Therefore, it conforms to the research design of this study and can meet the analysis requirements of this study.

The research region of this study is located in the suburbs of Shanghai, China, with a planning increment-type area, planning balance-type area, and planning reduction-type area, which can reflect the CLR of Shanghai, China. W District of Shanghai, China is located on the southern wing of the Yangtze River Delta, southwest of Shanghai, China. W District belongs to the CLR region, and is also an important region for Shanghai, China, to promote the rural revitalization strategy.

### 3.3. Data Source

The data in this study are village-level data in W District, Shanghai, China, from 2008 to 2020. The data used to divide the types of land use planning are derived from the Master Plan and General Land Use Plan of W District, Shanghai (2017–2035). The land-use data are extracted from the land-use status of each year with ArcGIS, including 124 administrative villages or residents’ committees of nine towns and one industrial zone. According to the Notice on Printing and Issuing Trial “Land Classification” (Land Resource Development No. 255, 2001) and the national standard Classification of Land Use Status (GB/T 21010-2017 instead of GB/T 21010-2007), the construction land type is summarized as industrial and mining storage land, urban residential land, rural residential land, commercial land, and other construction lands. Economic and social data from 2008 to 2020 are obtained from the Statistical Yearbook of W District. The commodity retail price index was used to adjust the retail sales of social consumer goods. Except for the 2020 retail price index, which is from China Premium Database, the retail price indexes for other years are from the 2020 Shanghai Statistical Yearbook. The rest of the value-based data are deflated using the consumer price index for 2008 as the base period to eliminate the effect of inflationary factors. Except for the 2020 consumer price index derived from the 2020 Shanghai Statistical Bulletin on National Economic and Social Development, the consumer price indexes in the other years all come from the 2020 Shanghai Statistical Yearbook.

### 3.4. Model Building

The DID model was first proposed by Ashenfelter and Card for assessing the impact of the Comprehensive Employment and Training Act on student income [75] and was later widely used in the fields of policy assessment [76,77,78]. This study adopted the DID model to empirically test the impact of SI on the economic development of NRRCL. The benchmark regression model is as follows:

*LnPergdp_it_* = *α_0_* + *β*_1_⋅*Policy_i_* + *β*_2_⋅*Post_t_* + *β*_3_⋅*Policy_i_*⋅*Post_t_* + *β*_4_⋅*Planning_i_* + *∑δ_k_*⋅*x_k_* + *λ_i_* + *θ_t_* + *ε_it_* (25)

In Equation (25), subscript *i* represents the administrative village or residents’ committee, subscript *t* represents the time, and *n* is the number of control variables. *LnPergdp* is the level of regional economic development of the administrative village or residents’ committee. *Policy* is the group dummy variable for the experimental group and control group. *Post* is the group dummy variable for the pre-policy and post-policy. *Planning* is the type of land-use planning. *λ_i_* is the individual-fixed effect, and *θ_t_* is the time-fixed effect. The interaction terms of *Policy* and *Post* are included in the model, that is, whether there is SI in the administrative village or residents’ committee in the current year. *β*_3_ is the core estimated variable, reflecting the treatment effects concerned in this study. According to the research assumptions, *β*_3_ is expected to be significantly negative, *β*_4_ is the influence coefficient of the type of land-use planning, *x_k_* is the other control variables affecting the economic level of NRRCL (*k* ∈ 1, 2, …, *n*), and *δ_k_* is the influence coefficient of the control variables.

The basic premise of using the DID method is the parallel trend assumption. For the parallel trend test, the interaction terms of *Policy* and *Post* are added to the regression model as the explanatory variables. Each interaction term serves as the core explanatory variable, and its estimated coefficients indicate the size of the difference between the groups in each year. The dynamic trend test was used to check whether the benchmark regression model met the parallel trend hypothesis [79]:

*LnPergdp_it_* = *α_0_* + *β*_1_⋅*Policy_i_* + *β*_2_⋅*Post_t_* + *∑η_t_*_−2014_⋅*Policy_i_*⋅*Post_t_* + *β*_4_⋅*Planning_i_* + *∑δ_k_*⋅*x_k_* + *λ_i_* + *θ_t_* + *ε_it_* (26)

In Equation (26), *t* ∈ 2008, 2009, …, 2020, the control variables and fixed effects (FE) are set up to be consistent with Equation (25). If the benchmark model satisfies the parallel trend assumption, the coefficients of *η*_−6_, *η*_−5_, *η*_−4_, *η*_−3_, *η*_−2_, and *η*_−1_ should not be statistically significant.

### 3.5. Variable Selection and Index Measurement

The dependent variable is *LnPergdp*, measured by the log value of the actual per capita GDP. As for the robustness test, the log value of the actual per unit of administrative division area GDP (*LnLandgdp*) and the log value of the actual GDP (*LnGdp*) were also adopted for measurement. The output value of the primary, secondary and tertiary industries in the town or industrial zone of the administrative village or the residents’ committee are respectively decomposed by the cultivated land, industrial and mining storage land, and commercial land to each administrative village or residents’ committee.

There were two core explanatory variables in this study. One of the core explanatory variables is SI, measured by *Policy⋅Post*. The other core explanatory variable is *Planning*. Learned from the existing literature, this study defined SI in combination with the actual situation in Shanghai, China. The measurement of spatial equity is measured based on the perspective of spatial accessibility [67]. Accessibility refers to the distance one is from one place to another [44] and is a tool used to measure equity [64]. Some studies considered spatial equity to mean equal access to basic public facilities, which can be measured using distance [64]. Fair location of various facilities also takes distance as an important consideration [80]. In addition to accessibility, there were also studies suggesting that the population represents the demand of a region for the number and type of services [65,66,67]. To reflect the relationship between the population of a region and the number of service opportunities provided, “land per capita” is a viable index [68]. This index was also used by urban planners to determine the scarcity of available land volumes for each service type based on the lowest service standards [67].

Thus, this study calculated the ratio of the actual per capita GDP of each administrative village or residents’ committee in 2020 and 2008 (*Ratio*):

*Ratio_i_ = Pergdp_i_*_,2020_*/Pergdp_i_*_,2008_ (27)

In Equation (27), *Pergdp_i_*_,2020_ and *Pergdp_i_*_,2008_ represent the actual per capita GDP of each administrative village or residents’ committee in 2020, 2008, respectively.

Then, *Policy* can be described as follows:

*Policy_i_ =* 1*, if Ratio_i_ <* 1; *Policy_i_ =* 0*, Otherwise* (28)

*Policy_i_* = 1 is the experimental group; that is, there is SI. *Policy_i_* = 0 is the control group; that is, there is no SI. The core logic for this treatment lies in that the principle of reduction in location selection in the process of CLR is “maximization of location disadvantage” [20], while the principle of the increment in location selection for the allocation of the saving quotas after CLR is “maximization of location advantage”. Therefore, under the control of the total amount and intensity of construction land, the originally scattered, inefficient, and seriously polluted construction land outside the concentrated construction region is located in a poor location with low efficiency and is allocated relatively less construction land after the reduction, which belongs to the experimental group of SI.

To test the reliability of the findings of this study, we used the ratio of the actual construction land output value of each administrative village or residents’ committee in 2020 and 2008 to construct the experimental group. We assigned the value “1” to administrative villages or residents’ committees with a ratio lower than “1”; this group was considered the experimental group regarding SI (*Policy*_2_).

To this end, we calculated the ratio of the actual per unit of administrative division area GDP of each administrative village or residents’ committee in 2020 and 2008 (*Ratio*_2_):

*Ratio*_2*,i*_*= Landgdp_i_*_,2020_*/Landgdp_i_*_,2008_ (29)

In Equation (29), *Landgdp_i_*_,2020_ and *Landgdp_i_*_,2008_ represent the actual per unit of administrative division area GDP of each administrative village or residents’ committee in 2020 and 2008, respectively. *Policy*_2_ can be expressed as follows:

*Policy*_2,*i*_*=* 1*, if Ratio*_2,*i*_
*<* 1; *Policy*_2,*i*_
*=* 0*, Otherwise* (30)

*Policy*_2,*i*_ = 1 is the experimental group, while SI. *Policy*_2,*i*_ = 0 is the control group, without SI.

In terms of *Planning*, according to the established literature [1], the research region is divided into three types. The first type is the change of the average planned construction land area between 10% and 50%, which is called the planning increment-type area (*Planning_I*), including Town AA (14.07%), Industrial Zone BB (19.38%), and Town CC (21.82%). The second type is the change of the average planned construction land area between −10% and 10%, which is called the planning balance-type area (*Planning_B*), including Town DD (−2.21%), Town EE (3.19%), Town FF (3.21%) and Town GG (6.09%). The third type is the change of the average planned construction land area between −10% and −50%, which is called the planning reduction-type area (*Planning_D*), including Town HH (−26.83%), Town II (−11.89%), and Town JJ (−15.00%). In the model, the *Planning_I* group is the reference for regression analysis.

An important prerequisite for the DID approach is the parallel trend. The second prerequisite is that the experimental group should satisfy the random distribution characteristic. To avoid bias in the evaluation results by nonrandom allocation, control variables were added to the benchmark regression model to exclude the interference of nonrandom allocation factors with the results [77,81]. Moreover, the precise estimation of DID methods needs to ensure that the empirical model is disturbed as little as possible by missing variables. Thus, we excluded the interference by methods including controlling the relevant variables and fixed effects of multiple dimensions. For the control variables, we controlled the level of total social expenditure (*Sales*). In addition, the agricultural population (*Pagri*), the level of urbanization (*Rurban*), and the location condition of township (*Dist*) are also included. The specific interpretation and indicator measures of each variable of the main model are shown in Table 1.

Descriptive statistics for the main variables are presented in Table 2. According to the theoretical analysis, it can be found that under the control of the total amount and intensity of construction land, the construction land tends to concentrate in the dominant region. According to Table 2, about 31.45% of administrative villages or residents’ committees have SI, so CLR realizes the development of NIRCL through the reduction of construction land of NRRCL, which causes SI. In the research interviews, government staff and local residents of NRRCL felt that SI affected local employment, local house rental income, and household income, thus affecting the enthusiasm of CLR under the current policy. H1 was verified.

## 4. Results

### 4.1. Benchmark Regression Results

Before regression analysis, we adopted multicollinearity to test the variables. Variance inflation factors (VIF) are widely used in multicollinearity tests. Generally, VIF values greater than 10 indicate severe multicollinearity [82]. In this study, all VIF values were much lower than 10. This indicates that there is no severe multicollinearity between the variables.

The benchmark regression results for SI affecting the economic development of NRRCL are shown in Table 3. In Column (1) of Table 3, no control variables, time-fixed effects, or individual-fixed effects were added. In Columns (2) and (3), time-fixed effects and individual-fixed effects were further controlled. From Columns (1)–(3), the coefficient of *Policy⋅Post* is significantly negative at the significance level of 1%, indicating that SI has a significant negative effect on the LnPergdp of NRRCL. In Column (4), the types of land-use planning variables were added to analyze the impact of different land planning types on the economic development. It can be found that the coefficients of both Planning_B and Planning_D are significantly negative at the significance level of 1%, indicating that land planning significantly reduces the LnPergdp of planning balance-type area and planning reduction-type area compared with planning increment-type area. In Column (5), the control variables were further added, and the study conclusion is consistent with Columns (1)–(4).

From Column (5) of Table 3, the coefficient of Policy⋅Post is significantly negative at the significance level of 1%, indicating that SI has a significant negative effect on the LnPergdp of NRRCL. Studies have found that in Jinshan District, Fengxian District, and Qingpu District of Shanghai, China, the tax amount per unit area in the development zone is three times or even five times that of outside the development zone [20]. Due to its comparative advantages and high output efficiency, NIRCL is the focus of construction land allocation. As mentioned above, due to structural optimization, the marginal space use tendency of decision-makers under the control of the total amount and intensity of construction land is for NIRCL, resulting in the lack of construction land allocation in NRRCL, which makes it difficult to realize the land development rights or play the advantage of backwardness, leading to the slow development of NRRCL. Therefore, H2 is verified.

As can be found in Column (5) of Table 3, the coefficients of both Planning_B and Planning_D are significantly negative, indicating that land planning significantly reduces the LnPergdp of planning balance-type area and planning reduction-type area compared with planning increment-type area. Compared with planning increment-type area, the other two types of areas lack comparative advantages in non-agricultural industry development. Moreover, land planning allocates more resources to planning increment-type area, resulting in the slow development of planning balance-type areas and planning reduction-type areas (the construction land quotas of the planning balance-type area are basically for local use; so, the direction of its role may be close to the planning reduction-type development region, and also has the characteristics of planning increment-type area. The quota cost of planning increment-type area using planning reduction-type area is rising, and the coordination problem exists across regions. A planning balance-type area can decrease and increase quota coordination itself, and thus has a low cost). Therefore, H3 is verified.

The influence coefficient of SI on the LnPergdp of NIRCL is 0.6812 and that of NRRCL is −0.0332 (0.6812–0.7144; see Column 5 of Table 3). Hence, SI significantly reduces the economic development level of NRRCL and promotes the development of NIRCL. In other words, regarding SJ of CLR in suburbs, the off-site realization has comparative advantages over in situ realization. H4 is therefore verified.

### 4.2. Parallel Trend Test with Dynamic Effect Estimates

**Figure 2:**
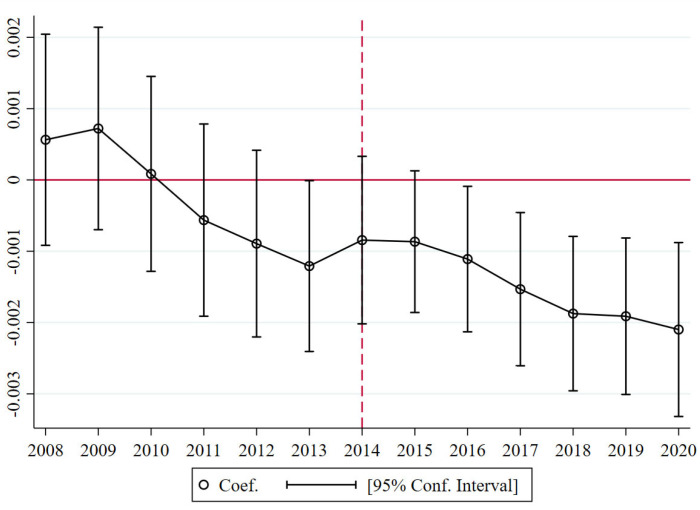
Parallel trend test chart.

We created the parallel trend chart according to the coefficient estimates and their confidence intervals of Equation (26). The results are shown in Figure 2. There was no significant downward trend from 2008 to 2013, whereas 2014 and later present a significant downward trend, indicating that no other important events occurred previously and thus no bias was observed for the estimated results. The coefficient values of the differences between the groups were not significantly different from zero before the impact, thus indicating that there was no significant difference in economic development between the treatment and control groups after controlling for other related factors, verifying the reliability of this study.

### 4.3. Replacing the Dependent Variable

We tested the robustness of the benchmark regression results by replacing the dependent variables and analyzing the differential effect of SI on actual per unit of administrative division area GDP and actual per capita GDP. Because the administrative division area of each administrative village or residents’ committee is unchanged, but the population can move freely, this study analyzed the change law of actual per capita GDP and actual per unit of administrative division area GDP. During the CLR process, if the population can be transferred along with the construction land quotas, it will help to not reduce or even increase the welfare level of the existing population in NRRCL (that is, the population decreases, GDP decreases, and per capita GDP increases, which coexist in NRRCL).

The regression estimation was repeated using LnLandgdp as the dependent variable. The results are shown in Columns (1)–(3) of Table 4. It can be seen that the coefficient of Policy⋅Post is negative and has passed the significance level test of 1%. The regression results remained robust after adding the control variables. This indicates that SI has negative effects on the LnLandgdp of NRRCL. Moreover, the coefficients of Planning_B and Planning_D are significantly negative, implying that land planning significantly reduces the LnLandgdp of planning balance-type area and planning reduction-type area compared with planning increment-type area.

This study also uses LnGdp as the dependent variable to repeat the regression estimation. Results are shown in Columns (4)–(6) of Table 4. The coefficient of Policy⋅Post is negative and has passed the significance level test of 1%. This indicates that the influence of SI on the LnGdp of NRRCL is significantly negative. The regression results remained robust after adding the control variables, and the coefficients of Planning_B and Planning_D are significantly negative. Therefore, the land planning significantly reduces the LnGdp of planning balance-type area and planning reduction-type area compared to planning increment-type area. Thus, the research conclusion of this study is shown to be robust.

Further, by comparing the coefficients of Policy⋅Post (−0.7144) of Column (5) of Table 3, the coefficients of Policy⋅Post (−0.5932) of Column (3) of Table 4, and the coefficients of Policy⋅Post (−0.5960) of Column (6) of Table 4, we can find that, from the “per capita” point of view, SI has a more significant negative impact on NRRCL, with the influence coefficient of −0.7144. Moreover, the influence of SI on the LnLandgdp and LnGdp of NRRCL shows rather close changes in the same direction, with the influence coefficients of −0.5932 and −0.5960, respectively. If the output is assumed to be positively proportional to the construction land area, the total output of NRRCL decreases due to the decrease in construction land area. If the population can be transferred to the NIRCL, that is, with a synchronous—or even greater—reduction, the per capita output will not decline or even increase. This shows that during the CLR process, with the transfer of land development rights in NRRCL, the population of NRRCL has not been transferred well, which has caused a decline in the social welfare of the residents of NRRCL.

The impact of SI on LnGdp is between LnPergdp and LnLandgdp, and the SI’s impact on LnGdp is similar to the impact on LnLandgdp. The area of administrative divisions is unchanged, but the agricultural output value is relatively low, mainly in non-agricultural industries. According to the 2021 Statistical Yearbook of W District*,* the total agricultural output value accounts for only 2.08% of the regional GDP. Therefore, the transfer of land development rights is unfavorable to the development of NRRCL, and some population transfer to NIRCL is necessary along with the construction land quotas. Therefore, H5 is verified.

Columns (3) and (6) of Table 4 show that the coefficients of Planning_B and Planning_D are significantly negative, indicating that land planning significantly reduces the LnLandgdp and LnGdp of planning balance-type area and planning reduction-type area compared to planning increment-type area. Due to the influence of land-use planning, the agricultural functions of planning balance-type area and planning reduction-type area can be strengthened. Land development rights are also limited, thus restricting the economic development of these two types of areas. Therefore, the research conclusion of this study is robust.

### 4.4. Impact of Testing the Definition of Treatment Variables on the Benchmark Regression Results

A new treatment variable was constructed based on the actual per unit of administrative division area GDP to test whether the treatment variables were defined with an effect on the benchmark regression results. The regression results are shown in Table 5. Columns (3), (6), and (9) of Table 5 show that the estimated coefficient of Policy_2_⋅Post is still significantly negative, and the benchmark regression results are not fundamentally changed.

In Columns (3), (6), and (9) of Table 5, the coefficients of Planning_B and Planning_D are significantly negative, indicating that land planning significantly reduces the output level of planning balance-type area and planning reduction-type area. Compared with planning increment-type area, the economic development level of planning balance-type area and planning reduction-type area is relatively low.

By comparing the coefficients of Policy_2_⋅Post (−0.6991) in Column (3) of Table 5, Policy_2_⋅Post (−0.6851) in Column (6) of Table 5, and Policy_2_⋅Post (−0.6892) in Column (9) of Table 5, we can find that from the “per capita” point of view, SI has a more significant negative impact on NRRCL, with the influence coefficient of −0.6991. Moreover, the influence of SI on the LnLandgdp and LnGdp of NRRCL shows rather close changes in the same direction. The influence coefficients were −0.6851 and −0.6892, respectively. Therefore, the research conclusion of this study is robust.

## 5. Discussion

This study analyzed the influence of SI on the economic development of NRRCL from the perspective of SI caused by the restriction of realization of land development rights and the advantages of backwardness. Essentially, CLR is the redistribution of land development rights under the constraints of planning, which is a kind of land consolidation [1]. In the suburbs, CLR was introduced as an innovative use of land consolidation wherein continued development is controlled by a designated total amount of construction land. Lewis’s theory of binary economic structure [9] assumes that urban and non-agricultural industrial land is satisfied and non-constrained by the process of transferring rural surplus labor force to urban regions and non-agricultural industries. However, in a populous country such as China, the growth of construction land is strictly controlled to protect the cultivated land and the ecological environment; hence, the unlimited expansion of construction land to meet the demands of development no longer exists [14,83,84]. Therefore, the development model of controlling the total amount of construction land is being explored in the economically developed regions of China.

In this study, five research hypotheses are proposed through theoretical analysis and empirically tested using the DID model, taking Shanghai’s W district as an example. This study enriches the empirical study of the impact of SI. Economic policies are well-intentioned, but there may be consequences in the process of policy implementation that undermine the development rights of disadvantaged regions. This study reveals the importance of protecting the development rights and interests of disadvantaged regions in the process of economic development. CLR has promoted regional economic development as a whole [1,15], but for NRRCL, it is difficult to bring into play the advantages of backward development, and thus development dilemmas arise. According to land development rights theory, CLR reclaims the inefficient construction land into cultivated land or ecological land, which is essentially a change of land use. The core of CLR lies in the transfer of land development rights, which affects the degree of SJ realization of NRRCL [15]. Land is the most critical factor in the restriction and development of the local economy; determining the use and redistribution of land is the most complex, technical, and important stage of land consolidation [85]. SI affects the economic development of NRRCL via two aspects. From the perspective of CLR objects, the reduction aims to reduce the construction land outside centralized construction regions that are inefficient, scattered, seriously polluted, or out of compliance with urban development planning. Hence, NRRCL with more inefficient construction land is naturally assigned more CLR tasks [20]. Meanwhile, from the perspective of CLR saving quota allocation, the saving quotas are allocated more often to the regions with good locations.

For the economic development of underdeveloped regions, it is necessary to provide space to support advanced enterprises and new populations and improve the backward production relations based on the advanced productive forces. If the development space is limited or even reduced, the advantage of backwardness cannot be played, and the advanced productive forces and relations cannot be realized, either. Developing regions have no chance to achieve their advantage of backwardness and are always in a disadvantaged position. For the sake of rural revitalization, the development of NRRCL needs to be given sufficient attention. Consistent with the established literature [1], this study similarly illustrates the importance of protecting the development interests of NRRCL. In addition, from the perspective of the types of land-use planning, this study found that the planning reduction-type area belongs to the damaged party of CLR. This is consistent with existing research [1]. Regarding SJ of CLR in the suburbs, the off-site realization has comparative advantages over in situ realization; this is consistent with existing research [1,15]. Residents of the net reduction regions should move with the transfer of balanced quotas to ensure that these people enjoy the benefits of the net increment regions [15]. This study also found that the population transfer with the construction land quota plays an important role in alleviating the impact of SI on NRRCL’s economic development.

## 6. Policy Implications and Conclusions

### 6.1. Policy Implications

SI restricts the economic development level of NRRCL. In the process of CLR, the problem of SJ realization of NRRCL should be solved to promote the economic development of NRRCL, including four main aspects:

(1) Improve the development capacity of non-agricultural industries by increasing the proportion of construction land quota allocation in NRRCL. In the allocation of the saving quotas formed through CLR, it is essential to consider factors such as regional differences in resource endowment and economic and social development and to address development opportunities of backward regions. Securing a lasting and stable income for rural collective economic organizations is an important challenge in CLR regions. Due to the industrial disadvantage of NRRCL, such as poor operating income and lack of comparative advantage, using excessive quotas for the development of industry and commerce in this region does not conform to the development law of urbanization. Therefore, in terms of CLR policy, a certain proportion of the CLR saving quotas should be allocated to the strengthening of NRRCL function to meet the needs of marketization. It will help to promote the economic and social development of NRRCL through rural tourism and urban sightseeing agriculture [86]. Under the control of the total amount and intensity of construction land, when the construction land area cannot meet the development of NRRCL, the construction land utilization efficiency of NRRCL can be promoted by increasing the floor area ratio, thus, guaranteeing the development rights and interests of NRRCL.
(2) Increase the one-time or long-term compensation of NIRCL to NRRCL to increase its income source. Urban–rural integration is the ideal urban–rural relationship for balancing urban and rural development [87]. When NIRCL uses the construction land quotas of NRRCL, it contributes back to NRRCL by means of capital and technology. Higher-level governments, such as municipal and district governments, should coordinate the benefit distribution between NRRCL and NIRCL and dredge the population transfer mechanism from NRRCL to NIRCL. The government should promote the concentration of population, residence, and employment in cities and towns, protect the employment, residence, social security, and collective assets rights and interests of NRRCL, and make sure the realization of the collective assets rights and interests of rural residents that transfer to the cities through financial support, so as to reach a win-win stage for both NRRCL and NIRCL while promoting the integrated development of urban and rural regions.
(3) Increase the residence and employment transfer of the NRRCL population to NIRCL to maintain the welfare of the NRRCL population. We should promote the transfer of the NRRCL population to NIRCL through a centralized residence, and optimize the living conditions of NRRCL residents so that NRRCL residents can share the benefits of urbanization. We should create more jobs through NIRCL’s industrial agglomeration and reduce the negative impact of SI on NRRCL employment by absorbing NRRCL residents into NIRCL employment.
(4) Attach importance to the economic development of planning reduction-type areas. Planning reduction-type areas have comparative advantages in developing non-agricultural industries with rural characteristics and urban sightseeing agriculture; therefore, policy support should be given to make them develop and grow. However, agriculture and rural industries have limited growth potential when compared to NIRCL’s non-agricultural industries. Therefore, it is important to transfer the NRRCL population to NIRCL and to realize a complete outmigration of the population in NRRCL [1]. To this end, relevant policies need to be formulated. The “three no change policies” in the current policy do not meet the needs of development and are not conducive to the absolute reduction of NRRCL population. There is no systematic policy designed for population transfer, and the existing policies are incomplete and uncoordinated with a small force. Therefore, further in-depth research is required on these areas.

### 6.2. Conclusions

Based on the theoretical research, Shanghai’s CLR policy was taken as a quasi-natural experiment. The DID model was adopted to empirically study the effects of SI on the economic development of NRRCL. The main conclusions of this study are as follows:

(1) Due to the difference between the marginal space reduction tendency of construction land and the marginal space use tendency of land development rights, the regional difference policy of CLR in economically developed regions promotes the transfer of land development rights from NRRCL to NIRCL, producing SI. (2) SI restricts the advantage of backwardness of NRRCL, thus limiting the economic development level of NRRCL. (3) Land-use planning has a negative impact on the economic development of planning reduction-type areas. (4) NIRCL has a comparative advantage over NRRCL in solving the development problems; in other words, the off-site realization of SJ in the CLR process has a comparative advantage. (5) As the NRRCL population is not fully transferred to NIRCL, the impact of SI on the per capita output of NRRCL is far greater than the average land output and total output. Therefore, in the implementation process of CLR policy, the SI problem should be solved to realize the economic development of NRRCL.

## Figures and Tables

**Table 1:** Description of main model variables.

Variable Type	Variable Name	Variable Code	Description
Dependent variable	The level of regional economic development	LnPergdp	The log value of the actual per capita GDP of each administrative village or residents’ committee
Explanatory variables	Grouping dummy variable	Policy	Policy = 1 is the experimental group; Policy = 0 is the control group
	Policy implementation dummy variable	Post	Post equals 0 for every year before 2014; Post equals 1 for every year after 2013
	Spatial injustice	Policy⋅Post	The interaction term between Policy and Post
	Types of land-use planning	Planning_B	Is it a planning balance-type area? “Yes” = 1, “No” = 0
		Planning_D	Is it a planning reduction-type area? Yes” = 1, “No” = 0
Control variables	The level of total social expenditure	Sales	Logarithm of the total society retail sales of the town or industrial zone where each administrative village or residents’ committee is located
	The agricultural population	Pagri	The agricultural population of the town or industrial zone where each administrative village or residents’ committee is located
	The level of urbanization	Rurban	The urbanization rate of the town or industrial zone where each administrative village or residents’ committee is located
	Location condition of township	Dist	Logarithm of the distance from the administrative village or residents’ committee to the District government station

**Table 2:** Descriptive statistics of the main variables.

Variable	Unit	Obs	Mean	Std. Dev.	Min	Max
Gdp	Million CNY	1612	228.2107	277.7197	3.6061	1460.7600
Pergdp	10,000 CNY/person	1612	8.1974	17.6135	0.1463	455.3275
Landgdp	10,000 CNY/mu	1612	3.2667	3.6000	0.0667	19.2000
Post	–	1612	0.5385	0.4987	0.0000	1.0000
Policy	–	1612	0.3145	0.4645	0.0000	1.0000
Planning_B	–	1612	0.4919	0.5001	0.0000	1.0000
Planning_D	–	1612	0.2661	0.4421	0.0000	1.0000
Rurban	%	1612	61.7566	13.2478	28.9500	95.2000
Pagri	10,000 persons	1612	1.8473	0.8564	0.2795	3.3061
LnSales	CNY	1612	21.4056	0.5276	20.2584	22.3620
LnDist	Meter	1612	9.1721	0.4932	8.1551	9.8684

Note: 1 mu ≈ 0.067 hectares. “–” means that the variable has no unit.

**Table 3:** Benchmark regression results.

Variable	Dependent Variable: *LnPergdp*				
	(1)	(2)	(3)	(4)	(5)
Post	0.3745 ***	0.4778 ***	0.4778 ***	0.4778 ***	0.6812 ***
	(0.0639)	(0.1532)	(0.0574)	(0.0574)	(0.1983)
Policy	−0.1380	−0.1380	2.3026 ***	0.6090 **	2.7078 ***
	(0.0950)	(0.0953)	(0.2618)	(0.2786)	(0.2831)
Policy⋅Post	−0.7314 ***	−0.7314 ***	−0.7314 ***	−0.7314 ***	−0.7144 ***
	(0.1280)	(0.1284)	(0.0405)	(0.0405)	(0.0455)
Planning_B				−2.7810 ***	−3.0446 ***
				(0.1466)	(0.1816)
Planning_D				−1.6936 ***	−2.5947 ***
				(0.1467)	(0.3591)
Control variables	N	N	N	N	Y
Time FE	N	Y	Y	Y	Y
Individual FE	N	N	Y	Y	Y
Observations	1612	1612	1612	1612	1612
R-squared	0.0703	0.0735	0.9142	0.9142	0.9148

Note: Robust standard errors in parentheses. *** and ** indicate statistical significance at 1% and 5%, respectively. (1), (2), …, and (5) represent the first column to the fifth columns, respectively.

**Table 4:** Regression results when replacing the dependent variable.

Variable	Dependent Variable					
	LnLandgdp			LnGdp		
	(1)	(2)	(3)	(4)	(5)	(6)
Post	0.5103 ***	0.5103 ***	0.3319 **	0.5256 ***	0.5256 ***	0.3653 **
	(0.0474)	(0.0474)	(0.1608)	(0.0485)	(0.0485)	(0.1629)
Policy	1.6720 ***	0.5765 ***	2.0747 ***	1.3051 ***	0.6367 ***	2.8508 ***
	(0.1163)	(0.1306)	(0.1061)	(0.1159)	(0.1308)	(0.1071)
Policy⋅Post	−0.6288 ***	−0.6288 ***	−0.5932 ***	−0.6311 ***	−0.6311 ***	−0.5960 ***
	(0.0325)	(0.0325)	(0.0334)	(0.0327)	(0.0327)	(0.0336)
Planning_B		−2.3242 ***	−2.7324 ***		−2.2417 ***	−2.6408 ***
		(0.1007)	(0.1223)		(0.1019)	(0.1238)
Planning_D		−1.0955 ***	−2.3466 ***		−0.6684 ***	−1.9677 ***
		(0.0748)	(0.2161)		(0.0755)	(0.2183)
Control variables	N	N	Y	N	N	Y
Time FE	Y	Y	Y	Y	Y	Y
Individual FE	Y	Y	Y	Y	Y	Y
Observations	1612	1612	1612	1612	1612	1612
R-squared	0.9364	0.9364	0.9376	0.9461	0.9461	0.9471

Note: Robust standard errors in parentheses. *** and ** indicate statistical significance at 1% and 5%, respectively. (1), (2), …, and (6) represent the first column to the sixth columns, respectively.

**Table 5:** Regression results after redefining the treated variable.

Variable	Dependent Variable								
	LnPergdp			LnLandgdp			LnGdp		
	(1)	(2)	(3)	(4)	(5)	(6)	(7)	(8)	(9)
Post	0.4242 ***	0.4242 ***	0.7639 ***	0.4918 ***	0.4918 ***	0.4693 ***	0.5073 ***	0.5073 ***	0.5039 ***
	(0.0631)	(0.0631)	(0.2075)	(0.0457)	(0.0457)	(0.1603)	(0.0467)	(0.0467)	(0.1624)
Policy_2_	−0.9662 ***	−2.6598 ***	0.0295	−1.1462 ***	−2.2417 ***	0.1574	−1.4884 ***	−2.1568 ***	1.3080 ***
	(0.1294)	(0.1641)	(0.3452)	(0.0865)	(0.1062)	(0.2413)	(0.0878)	(0.1081)	(0.2434)
Policy_2_⋅Post	−0.7056 ***	−0.7056 ***	−0.6991 ***	−0.7173 ***	−0.7173 ***	−0.6851 ***	−0.7207 ***	−0.7207 ***	−0.6892 ***
	(0.0387)	(0.0387)	(0.0420)	(0.0348)	(0.0348)	(0.0376)	(0.0350)	(0.0350)	(0.0379)
Planning_B		−2.7810 ***	−2.9384 ***		−2.3242 ***	−2.5753 ***		−2.2417 ***	−2.4823 ***
		(0.1474)	(0.1827)		(0.0999)	(0.1207)		(0.1010)	(0.1222)
Planning_D		−1.6936 ***	−2.3205 ***		−1.0955 ***	−1.9342 ***		−0.6684 ***	−1.5517 ***
		(0.1516)	(0.3673)		(0.0755)	(0.2111)		(0.0763)	(0.2133)
Control variables	N	N	Y	N	N	Y	N	N	Y
Time FE	Y	Y	Y	Y	Y	Y	Y	Y	Y
Individual FE	Y	Y	Y	Y	Y	Y	Y	Y	Y
Observations	1612	1612	1612	1612	1612	1612	1612	1612	1612
R-squared	0.9101	0.9101	0.9109	0.9386	0.9386	0.9391	0.9479	0.9479	0.9484

Note: Robust standard errors in parentheses. *** indicates statistical significance at 1%. (1), (2), …, and (9) represent the first column to the ninth columns, respectively.

## Data Availability

Not applicable.
